# A Note Concerning the Action of Chloroform

**Published:** 1891-01

**Authors:** Dudley Wilmot Buxton

**Affiliations:** Member of the Royal College of Physicians, Administrator of Anæsthetists in University College Hospital, the Hospital for Women, Soho Square, and the London Dental Hospital.


AMERICAN JOURNAL

—OF—

DENTAL SCIENCE.

VOL. XXIV. BALTIMORE, JANUARY, 1891. NO. 9.

ARTICLE I.

### A NOTE CONCERNING THE ACTION OF CHLOROFORM.

BY DUDLEY WILMOT BUXTON, M. D., B. S. LOND.

Member of the Royal College of Physicians, Administrator of Anæsthetists in University College Hospital, the Hospital for Women, Soho Square, and the London Dental Hospital.

(*Continued from page 378.*)

We are now in a position to examine in more detail the work of the Chloroform Commission, not because its experiments present any great novelty or reveal any unfamiliar facts, but because the systematic record of its proceedings presents to us a convenient centre about which to collect the knowledge obtained during the past few years in this country on the Continent, and the United States. Part II.* deals with experiments made, of which no graphic record was taken. 268 dogs and 31 monkeys were killed with chloroform which was given, under various circumstances, such as fasting after heavy meals, in different

* Lancet, Jan. 18, 1890.

postures, with and without inhalers. The animals all died from stopping of respiration, and, according to the report, an interval of time, varying from two to six minutes elapsed before cardiac movement ceased. Slow and prolonged chloroformization, asphyxial conditions, and struggling seemed to lessen the interval between the cessation of breathing and stopping of the heart's movements. The method of testing the failure of respiration is not mentioned; auscultation, and, in some cases, the placing of a needle in the heart muscle, was used to test cardiac action; while, in cases of doubt, the chest was laid open. Attempts at resuscitation were made after natural respiration had failed; but, as a rule, failed when resorted to after an interval of 60 seconds, being usually successful after 30 seconds' interval. The figures are: In forty-four cases successful, after an average of 28·2 seconds after respiration had failed; thirty-eight cases, unsuccessful, after an average of 31·5 seconds. These facts are highly significant. We shall see that chloroform acts directly upon the heart muscle, and up to a certain point this action can be carried without grave results; beyond that point one of two things happens: failure gradually results whether respiration goes on or not, or the heart remains able to maintain the circulation, so long as a low tension persists, but as soon as reaction occurs, and the general vascular tone revives, the greater stress laid upon the heart leads to the utter extinction of its action, and cardiac syncope takes place. The occurrence of death in this last way accounts for fatalities, common enough, occurring as the patient, after a surgical operation, is beginning to come round. The animals in which the respiration failed before the heart were, none the less, killed by heart-syncope, for, whereas the respiratory failure resulted from poisoning of the respiratory centres in in the medulla concurrent, action upon the heart-muscle was going on, and, although perfect artificial respiration was performed, the heart-muscle, being poisoned, failed. To understand this, one has to be familiar with the following facts, which are daily noted in the physiological laboratory: that artificial respiration will keep a dog or monkey alive for hours, if the heart be not poisoned by chloroform even when the medullary centres are quite inactive; and, secondly, that dogs, especially, are difficult to save when once breathing has ceased, even though prompt resort be had to artificial respiration.

Again, the animals subjected to slow and prolonged chloroform, and those which struggled, incurred cardiac syncope most rapidly. Here, again, we find evidence of my contention. The prolonged action of the chloroform upon the heart led to the pathological changes which give rise to syncope while, in the struggling animals, a greater strain was imposed upon an already enfeebled heart, and hence early syncope obtained.

Turning to the experiments, the results of which were recorded by apparatus, we are informed that, in a certain number of cases, "the animal died accidentally before it was ready to be attached to the manometer" (used for taking blood pressure curves). These deaths, as was noticed above, may or may not have been due to primary heart failure.

The objects kept in view during these experiments were five in number:—

I.—To test suitability and safety of chloroform as an anæsthetic. To establish some comparison, experiments were made with ether and the A.C.E. mixture (presumably that of the Medico-Chirurgical Society Committee on Chloroform, 1864); but as the commission themselves admit the experiments were insufficient to afford a complete exposition of the action of these drugs, as a matter of fact the ether and A.C.E. experiments may be disregarded, for they are not only too few to be of the least value but were conducted in a very unsatisfactory manner.

II.—The effect of pushing the anæsthetics (*a*) to a dangerous degree, (*b*) until failure of respiration (*c*) to death.

III.—Modification in symptoms caused by anæsthetics when complicated by asphyxia; by pressure of other drugs the alkaloids, &c. These last experiments do not concern us.

IV.—To test the alleged liability to primary syncope, to secondary syncope, to reflex syncope through shock: the effect of fatty heart; hæmorrhage, &c.

V.—To test the effect of the anæsthetics on various animals, especially monkeys, as being the closest allied to human beings.

In eight experiments, animals were anæsthetized until death took place. The open method was employed and the anæsthetic pushed until respiration failed. It was noticed in these cases that the heart's action stopped about 3 min. 27.5 sec., on an average, after failure of respiration.

It will be noted that the animals were treated as human beings are treated, and it is not surprising they died in the most usual way, namely, from respiratory failure. All who have, like myself, had abundant opportunities of studying the behavior of dogs and monkeys under chloroform will recognize that the phenomena, somewhat elaborated by the commission, are very familiar, and even the relation of respiration to circulation will appear less novel than the report would seem to imply must be the case. In these experiments blood pressure fell, the heart, after primary stimulation, became feeble, the systolic contraction of the ventricles being gradually lessened.

The effect upon the heart of artificial respiration was experimented upon in the second series of cases. The average time elapsing between cessation of natural breathing and the failure of the heart was, in these experiments, 3 40-60 minutes (in one case 13 minutes,) and artificial respiration when commenced 52 seconds after the natural respiration had ceased (37 cases) resuscitation was effected. When tried in 46 cases after cessation of pulse, *i. e.*, 3 min. 5 sec. before cardiac stoppage (taking the average) it failed in 17, succeeded in 29. When tried after cessation of cardiac movements it failed. The results are easily understood when we recognize a fact, which it would appear the Commission does not accept, that the actual heart muscle is itself affected. McWilliam * has shown that chloroform produces a dilatation of the heart due to relaxation of its muscle. Indeed, as we know, both voluntary and involuntary muscles are rendered flaccid and relaxed by chloroform, it is not surprising to learn that the heart muscle also relaxes and dilatation results. When, however, only a small dose of chloroform is given, dilatation is followed upon withdrawal of the anæsthetic by contraction. But if a larger dose is taken the heart muscle fails to recover itself, the dose of chloroform taken is only such a one as permits of the heart's regaining its tone, *i. e.*, dilatation is succeeded by contraction. When, on the other hand, where artificial respiration fails to restore vital processes, the heart has dilated beyond the point when contraction can subsequently occur; the dose of chloroform has been too large. What is especially noticeable, both in these cases and those met with in actual practice, is that individuals differ so largely in their power of resisting or recovering from the chloroform cardiac syncope. But when we remember that the tonus of the heart muscle is itself subject to wide vicissitudes, depending upon the health or disease of the individual at the time, one readily understands how it is that a fatal dilatation of the myocardium may occur at one time and not at another.

Experimenting upon dogs to investigate the question, whether reflex inhibition of the heart's action can be produced under chloroform by "shock," the Commission formulated the deduction that no such reflex inhibition of the heart's action occurs. They add, "it is impossible to produce syncope from chloroform in dogs." Even accepting the experiments as sufficiently numerous to be of much

---

*See British Medical Journal, 1890, vol. ii., pp. 831, 948, &c.

value, I can only say that the Commission's results differ from those of other observers and from my own experience. I have insisted both in this paper and elsewhere that dogs show great immunity from primary syncope, the result of reflex cardiac inhibition; indeed, we find that shock is revealed far more in highly organized than in lowly organized animals. The more complex is the nervous system the greater is its liability to suffer from shock. In the animal kingdom mutilations of the most severe character are sustained without the individuals being apparently any the worse, but such an immunity is enjoyed less and less as we ascend in the animal scale, until we reach man in whom, as is well known, severe pain or even subjective emotions are capable of producing syncope. In dogs, cats, and monkeys, whether under chloroform, or not, a true faint is rarely met with as the result of a fright, or reflexly through irritation of a nerve, and so far the results arrived at by the Commission would seem to tally with my own; but only so far, for we now know that the heart is always directly affected by chloroform, the result revealing itself in dilatation to which syncope must eventually succeed should the continuance of the anæsthetic be carried beyond the point of final resiliency of the muscle. In man, however, reflex inhibition must be accepted as a proved fact, and my own experience in the matter leads me to assume a dogmatic position, for I have again and again witnessed profound interference both with circulation and respiration caused by reflex when a patient was under chloroform. Dragging upon the spermatic cord, its division in castration, manipulation of pelvic, or abdominal viscera in laparotomies, section of the optic nerve in enucleation, all give evidence of shock under chloroform and cannot fail to impress any careful observer with the grave danger to the heart when insufficient of the anæsthetic has been administered. In dental operations again, where the shock occasioned by laceration of branches of the fifth pair of nerves is out of proportion to the severity of the undertaking, there is an especial danger of primary cardiac failure through reflex inhibition. In the space at my command it is impossible to attempt to traverse all the tissues entered upon by the Hyderabad Commission, but upon the two main practical points to which I have addressed myself, viz., the Commission's contention that chloroform kills, not through the heart but through the respiration, and that shock does not, through reflex action, interfere with the heart under chloroform, I think we are bound to say that the Commission have failed to substantiate their position. And further, I would add that the so-called "conclusions" do not without grave reservation apply to human beings, while any attempt to make such an application is likely to lead to the most calamitous results by inducing a false confidence in the supposititious safety of the heart under chloroform, and by leading to a studied neglect of the signs of cardiac failure during administration of that anæsthetic. Much space and argument have been devoted by the Commission in the attempt to prove that the fall of blood pressure and weakened cardiac action from chloroform are themselves protective, inasmuch as they prevent a rapid circulation of the anæsthetic. Such a theory, however, must fall completely to the ground when it is recognized that the heart muscle and the arterial system are not a rigid series of tubes which can be regulated by a mechanism producing slowing or acceleration of their contraction, but are highly organized muscular tissue actually and directly affected by the chloroform circulating in the blood, and only capable of sustaining that injurious action to a certain point. The fall of blood pressure and the lessened cardiac action are phenomena of cardiac dilatation, and even if their occurrence prevented a fresh take in of chloroform they would, at the same time prevent a speedy elimination of the drug, such as would be necessary to ensure the after contraction of the heart muscle and maintenance of circulation. The indictment against chloroform, as it reads at present, is at least so severe as to make one regard it as an agent too capable of producing dangerous cardiac derangement, to be used save by those trained in its administration, and under circumstances when other anæsthetics cannot be used.

But when we find that even in the hands of the most skilled and wary chloroform kills, and, further, when we are assured upon very high authority that this lethal power of chloroform is due to changes in the heart muscle visible to the eye and demonstrable to all, we are led to still further narrow the field of its use, and to say that chloroform should only be employed when other anæsthetics are unable to be exhibited, and when all precautions against a fatality have been taken. Much capital has been made by those who appear to regard any animadversion upon chloroform as a personal reflection upon themselves, out of the supposed dangers of ether. In one of the less known medical journals a considerable stir was recently made about ether deaths, and "the many dangers of ether" were therein vaguely cited. A prolonged use of ether and chloroform has led me to the following conclusions :—

I. Ether is incomparably less dangerous. Among the many cases one meets with in hospital, especially where the patient, broken down by illness, privation, or debauchery, has to take an anæsthetic and manifests alarming symptoms, hovers as it were upon the confines of death, most perils arise during the administration of chloroform. And, further, when such occurrences take place under ether the recovery is less tardy and less doubtful than under chloroform.

II. The after effects of ether are not what they are so often represented to be. Bronchitis, pneumonia, or nephritis are most rare. I cannot recall one in my own practice, and when they do occur they are generally due to excessive and reckless use of the anæsthetic.

III. Chloroform is incontestably the less disagreeable anæsthetic to take, and this has led persons to use it as an introductory inhalation to ether, but the practice I believe is dangerous because the heart, weakened and relaxed by the chloroform, is less able with impunity to react to the stimulus of the ether. But the objectionable character of the ether is readily overcome when it is administered in combination with and succession to nitrous oxide, after the admirable method which we owe to the late Mr. Clover. This method also reduces the consumption of ether from a half-a-pint or more to one or two ounces for the duration of an ordinary operation. The importance of this becomes readily apparent when we remember that the lung and kidney trouble, to which reference is made above is dependent largely upon the quantity of ether which the patient inhales.

IV. It is easier to administer chloroform than ether, although anyone who will devote a very short time to the subject will readily enough master the use of Clover's small portable regulating inhaler. But, again, if it is easier to administer chloroform, it is still easier to kill with the use of this agent. In working with chloroform the point when urgent danger commences may be overlooked, but the contrary is the case with ether.

In conclusion, I may be permitted to say in what cases chloroform may be used. Accepting ether as our routine anæsthetic, except, of course, in dental surgery, in which nitrous oxide takes the first place, I should restrict chloroform to cases of prolonged operations about the jaw, tongue, mouth, larynx and trachea. In exceptional cases, operations for abdominal, thoracic, brain surgery may require chloroform, but I am dealing here rather with routine than exceptional cases. When chloroform has to be given, I believe free and even dilution of its vapour is of more importance than dribbling out the anæsthetic drop by drop, and so prolonging the period of "going off" and that of struggling. With care, the open method (that used by Sir Joseph Lister) answers well, but when, as in most of the operations named, the lint or towel is in the way of the operator, I find the use of the apparatus made by Krohne and Sesemann,* and modified from Dr. Junker's original model, secures a fairly uniform dilution and is handy and easily worked.

Summing up, it is asked, shall we retract our belief that chloroform is not a safe and appropriate anæsthetic for the minor operations of surgery, such, for example, as those of dental surgery? I would reply in the negative, because, as I have striven to show, not one iota of fresh evidence has been advanced by the recent experimental investigations into the action of chloroform showing its safety, while much has now accumulated proving its extreme danger as compared with other anæsthetics. Of course, it may be said one death in three thousand is at worse a small mortality, and this is so; but while one would let a patient incur that risk for a major operation, which would itself suggest to the patient the idea of risk, one should, I submit, hesitate before permitting the patient to incur the risk when presenting himself for an operation such as tooth extraction, which he and the rest of the world regard as a matter entailing no appreciable degree of danger.—*London Dental Record.*

---

ARTICLE II.

### TREATMENT OF ABSCESSES.

BY DR. FRANK OVERHOLSER, OF LOGANSPORT.

My interest in the subject of abscess has been considerably awakened by several cases of aggravated form of chronic abscess which have come under my observation. At the same time impressing me with the fact that the

* Shown at the Annual General Meeting of the British Dental Association at Exeter, 1890.

